# Spatial transcriptomics reveals regional characteristics of lupus nephritis in murine kidneys and immune response to prednisolone or Gancao Nourishing‐Yin decoction therapies

**DOI:** 10.1002/ctm2.70236

**Published:** 2025-02-16

**Authors:** Yanjuan Chen, Yong Chen, Xufa Yang, Zhenyou Jiang, Dongzhou Liu, Xiaoping Hong

**Affiliations:** ^1^ Department of Rheumatology and Immunology, Shenzhen Clinical Research Centre for Geriatrics The Second Clinical Medical College of Jinan University, Shenzhen People's Hospital Shenzhen China; ^2^ Department of Rheumatology and Immunology Affiliated Hospital of Zunyi Medical University Zunyi China; ^3^ Department of Rheumatology and Immunology The Second Clinical Medical College of Jinan University The First Affiliated Hospital of Southern University of Science and Technology, Shenzhen People's Hospital Shenzhen China; ^4^ Department of Microbiology and Immunology School of Medicine, Jinan University Guangzhou China

1

Dear Editor,

This study reveals the spatial immune landscape in Lupus nephritis (LN) mouse kidneys and evaluates the therapeutic effects of prednisolone (PDL) and Gancao Nourishing‐Yin decoction (GCNY).

LN is a major cause of end‐stage renal disease and increases mortality in systemic lupus erythematosus patients.^1^ Current treatments—glucocorticoid, cytotoxic drugs, and biological agents are effective but come with notable side effects.^2^ Traditional Chinese medicine (TCM) has recently shown promise in addressing LN‐related kidney injury.^3^ Our previous research found GCNY, a TCM formulation, demonstrated anti‐inflammatory and antioxidative effects,^4^, ^5^ suggesting that GCNY could be a complementary or alternative treatment for LN by modulating immune responses. Since glucocorticoids are considered a fundamental therapy,^2^ PDL was chosen as the control for GCNY treatment.

Spatial transcriptomics (ST) is a powerful tool for investigating the cellular dynamics and therapeutic effect in LN kidneys.^6^ In 2023, Tang et al. applied ST to LN patient kidney biopsies, identifying elevated *APOE* ^+^ monocytes that facilitated macrophage trafficking.^7^ In this study, we conducted ST on kidney tissues from lupus‐prone MRL/Lpr (Lpr) mice, Lpr mice treated with either PDL or GCNY, and MRL/Mpj (Mpj) control mice, using 10× Genomics Visium platform (Figure S1A). After bioinformatic analysis, totally 16 428 spatial spots and 32 285 genes were identified, exceeding 670 spots and averaging 3984 genes and 15 404 unique molecular identifiers (UMIs) per sample (Figure S1B). UMAP analysis showed effective integration across all groups (Figure S1C). Unsupervised clustering classified the spots into six regions 1–6, with region 2 having the fewest genes and UMIs, possibly due to its unique cell types (Figure 1A, Figure S1D). Region 1 was the largest, followed by regions 2 and 3 (Figure 1B).

**FIGURE 1:**
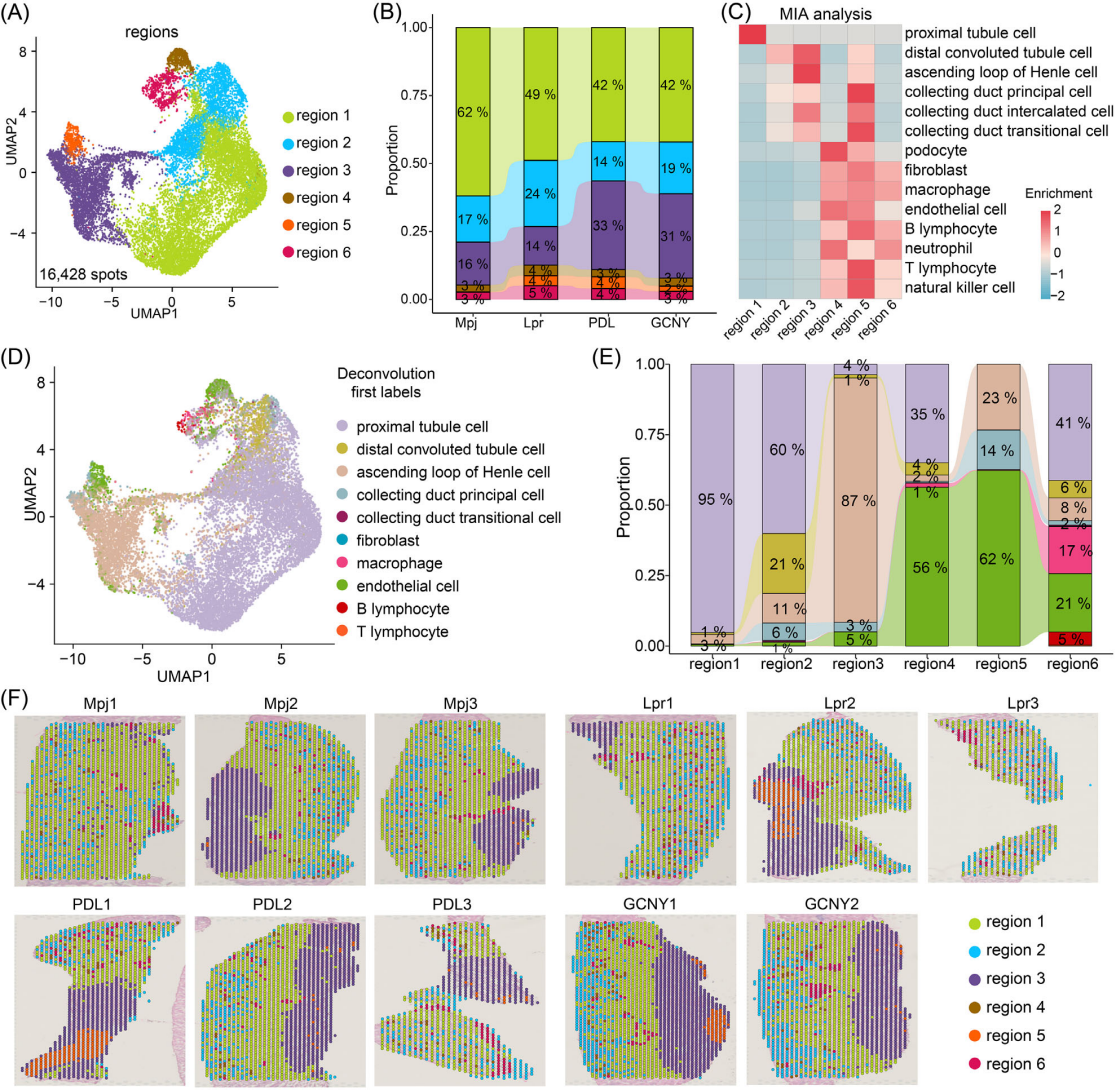
Spatial transcriptomic profiling of renal tissue in lupus‐prone mice. (A) The uniform manifold approximation and projection (UMAP) of 16 428 spatial spots, segmented into distinct regions based on unsupervised clustering. Each dot represents a spot, coloured by the inferred region. Each spatial spot on the slice was 55 microns in diameter and contained 2–10 cells. Kidney tissues were obtained from Mpj (n = 3), Lpr (n = 3), prednisolone (PDL)‐treated (n = 3), and Gancao Nourishing‐Yin decoction (GCNY)‐treated (n = 2). (B) Stacked bar plot illustrating the composition of each region across the groups. (C) Multimodal intersection analysis (MIA) heatmap depicting enrichment scores for scRNA‐seq‐identified cell types (GSE107585) across ST‐defined regions. The matrix elements are based on specific genes associated with each cell type. Red indicates higher enrichment, while blue indicates depletion. (D) UMAP plot showing cell subtype annotations for each spatial spot, determined by RCTD algorithm‐based deconvolution. Colours indicate cell types. (E) Stacked bar plot presenting the proportion of cell subtypes in each region. (F) Spatial images displaying region distributions on each tissue slice, with H&E‐stained sections shown underneath. Samples in the Mpj group are labelled Mpj1–Mpj3, with similar naming conventions for other groups.

Using the scRNA‐seq data as a reference (Figure S1E),^8^ the multimodal intersection analysis (MIA) indicated region 4, 5 and 6 mainly comprised podocytes, macrophages, neutrophils, T/B lymphocytes and natural killer cells (Figure 1C). The abundance of immune cells in regions 4–6 was highest in Lpr mice and reduced in PDL‐treated or GCNY‐treated mice (Figure 1B). Aligning with MIA results, spatial deconvolution analysis showed region 1 as predominantly proximal tubule cells and region 3 as mainly ascending loop of Henle cells (Figure 1D,E). Regions 4 and 6 exhibited the greatest variety of immune cell types, such as B lymphocytes and macrophages (Figure 1E). Based on spatial localization, regions 4 and 6 are scattered in the renal cortex, indicating their potential involvement in glomerular inflammation (Figure 1F). The characteristics of regions are detailed in Figure S2A–C. These results offer insights into the distinct spatial landscapes of LN kidney.

CellChat analysis showed that Lpr mice exhibited the highest level of cell‐cell interactions, which decreased with PDL or GCNY treatments (Figure S3A). The majority of interactions were concentrated in regions 4 and 6, areas rich in immune cells (Figure 2A, Figure S3B). In Lpr mice, complement signalling was notably elevated between these regions through Itgax‐Itgb2, Itgam‐Itgb2 and C3‐C3ar1 ligand‐receptor interactions (Figure S3C,D; Figure 2B,C). Similarly, transforming growth factor‐β (TGF‐β) signalling was upregulated via Acvr1‐Tgfbr1, Acvr1b‐Tgfbr2, and Tgfbr1‐Tgfbr2 receptor pairs. Region 6 also interacted with itself and region 4 through complement‐related ligand‐receptor pairs (Figure 2D,E). Interleukin‐1 (IL‐1) signalling was particularly directed from region 6 to regions 3 and 4 in Lpr mice. Additionally, chemokine signalling was elevated among regions 3, 4, and 6 through Ccl8‐Ccr5, Ccl3‐Ccr5 and Ccl5‐Ccr5 pairs (Figure 2D,E). The complement, TGF‐β, IL‐1 and CCL signalling were significantly diminished in treated kidneys. These pathways likely contribute to the anti‐inflammatory effects of PDL and GCNY in treating LN.

**FIGURE 2:**
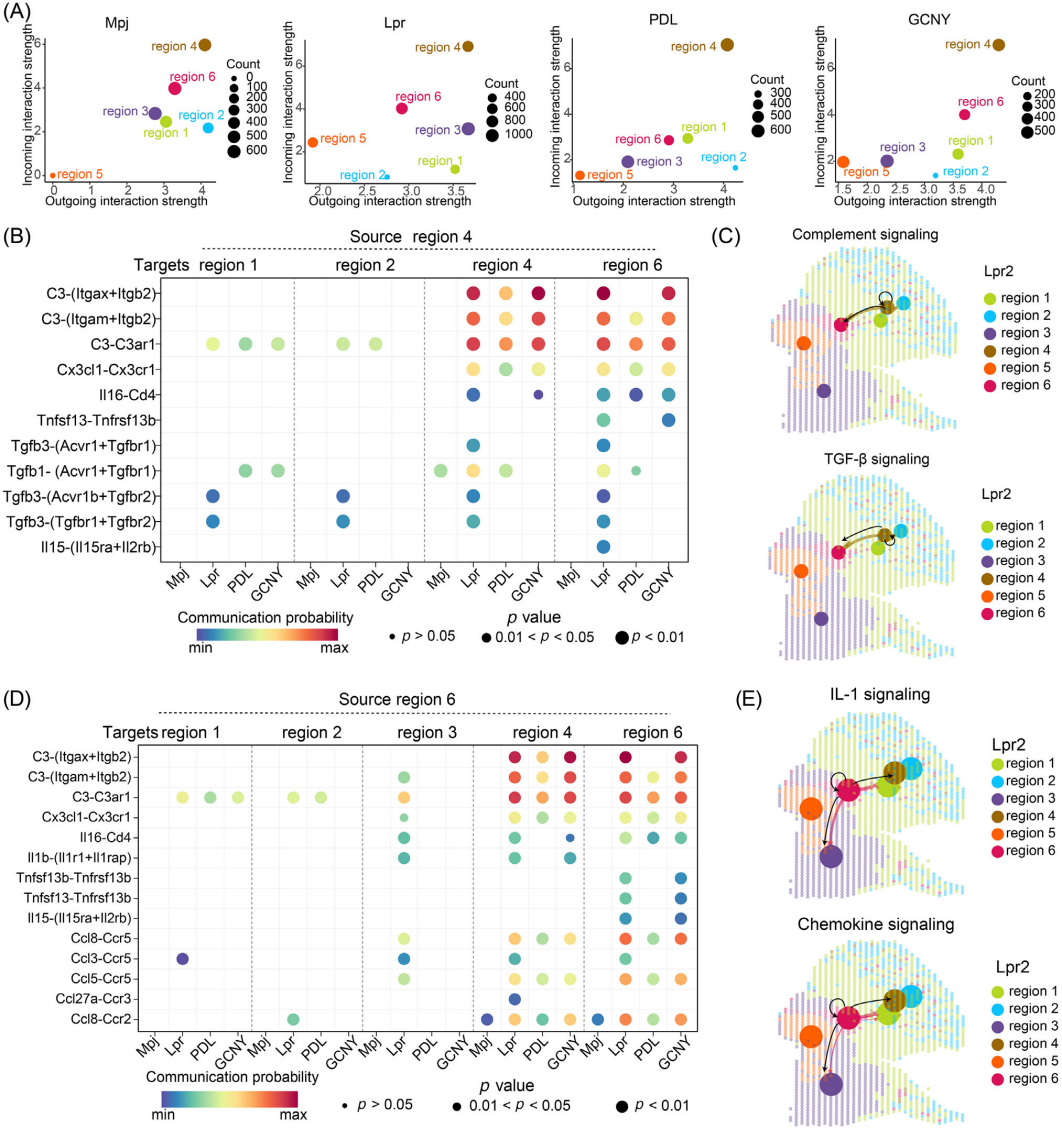
Comparative analysis of signalling patterns across groups. (A) Scatter plot illustrating the distribution of different regions based on the strength of efferent and afferent signalling interactions. (B) Dot plot comparing signalling patterns, with signal flow originating from region 4. Dot size corresponds to the contribution score from pattern recognition analysis, indicating the level of enrichment for each signalling pathway in the respective regions. (C) Spatial image depicting signalling pathways originating from region 4 in the Lpr2 slice. (D) Dot plot comparing signalling patterns, with signal flow originating from region 6. (E) Spatial image depicting signalling pathways originating from region 6 in the Lpr2 slice.

Macrophages are the key contributors to inflammation in LN.^7^ Deconvolution analysis showed that macrophages accounted for 2% of kidney cells in Lpr mice (Figure S1F). Scoring of macrophage‐specific genes confirmed their elevated presence in region 6 and region 4 (Figure S4A). Additionally, the score level was increased in LN kidneys, which significantly declined after treatment (Figure S4B,C). GSEA revealed suppression of lymphocyte and complement pathways in macrophages after PDL treatment (Figure S4C). In GCNY‐treated mice, lymphocyte activation was also downregulated but innate immunity pathways remained active (Figure S4D). However, immune‐related pathways were still activated in GCNY‐treated mice compared with PDL‐treated mice (Figure S4E).

In LN, the infiltration of B cells and plasma cells into renal tissues worsens inflammation.^1^, ^6^ Scoring of B cell‐related genes confirmed their elevated presence in region 6 and region 4 (Figure 3A). Compared to control mice, Lpr mice demonstrated a marked increase in B cell infiltration in the kidneys (Figure 3B,C). Treatment reduced the abundance scores of memory B cells and plasma cells (Figure 3B). Flow cytometry further confirmed a decrease in peripheral B220^+^ B cells and renal CD19^+^ B cells following PDL or GCNY therapy (Figure 3D,E). GCNY's immunosuppressive effect appears milder than PDL's. TCM, including our GCNY formula, tends to have a slower onset of action compared to the more rapid effects of chemical drugs like PDL.

**FIGURE 3:**
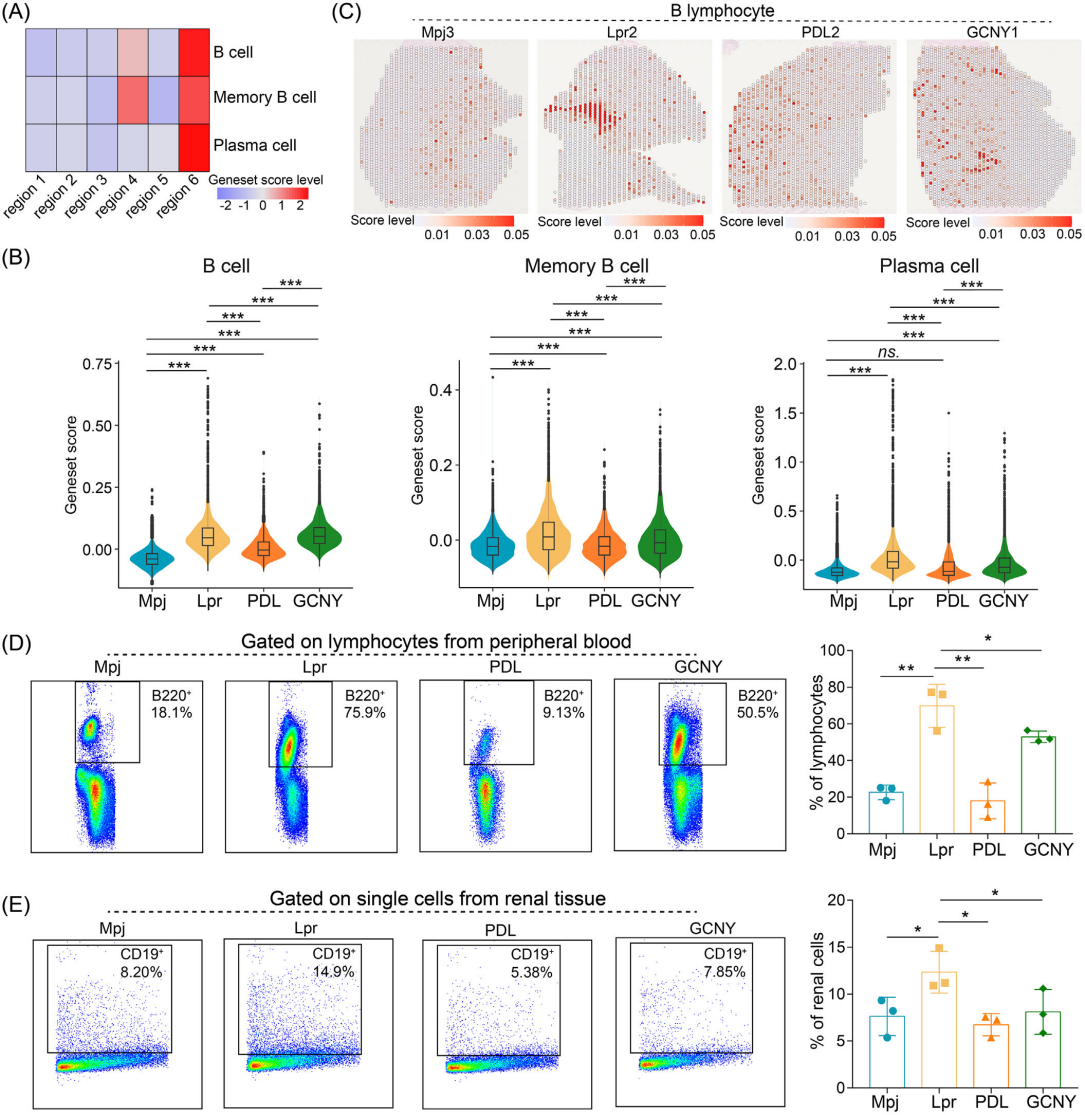
The characteristics of B lymphocyte across groups. (A) Heatmap showing the signature score levels for B cells, memory B cells, and plasma cells across regions. Red indicates higher score levels. (B) Violin plots displaying signature score levels for B cells, memory B cells, and plasma cells across groups, with percentiles indicated by the inside box plots (25%, 50% and 75%). (C) Spatial images showing B lymphocyte enrichment scores in tissue slices, with red indicating higher enrichment. (D) Flow cytometry scatter plots and bar plot showing the proportion of B220^+^ cells in peripheral blood from each group. (E) Flow cytometry scatter plots and bar plot showing the proportion of CD19^+^ cells in renal tissues from each group. Comparisons were made using the two‐sided unpaired Wilcoxon test (*p < 0.05, **p < 0.01).

Additionally, signatures scores for T cells and natural killer cells were highest in region 6 (Figure 4A). These score levels were also declined post‐treatment; however, the anti‐inflammatory effects of GCNY were less pronounced than those of PDL (Figure 4B,C). Previous research has indicated that immune cell clusters, identified as tertiary lymphoid structures (TLS), progressively form in LN kidneys.^9^ Our current study recognized TLS enriched with immune cells in the Lpr2 slice, which showed elevated enrichment scores for macrophages, B cells, and T cells (Figure 4D). Cells in the TLS expressed high levels of markers for leukocytes (*Ptprc*), T cells (*Cd3e*, *Cd4*), B cells (*Ms4a1*), plasma cells (*Cd79a*), macrophages (*Cd68*), and dendritic cells (*Itgax*).^7^, ^8^, ^10^ On contrast, low expression of *Cd8a* suggested the TLS in LN kidney tissue contained few CD8^+^ T cells.

**FIGURE 4:**
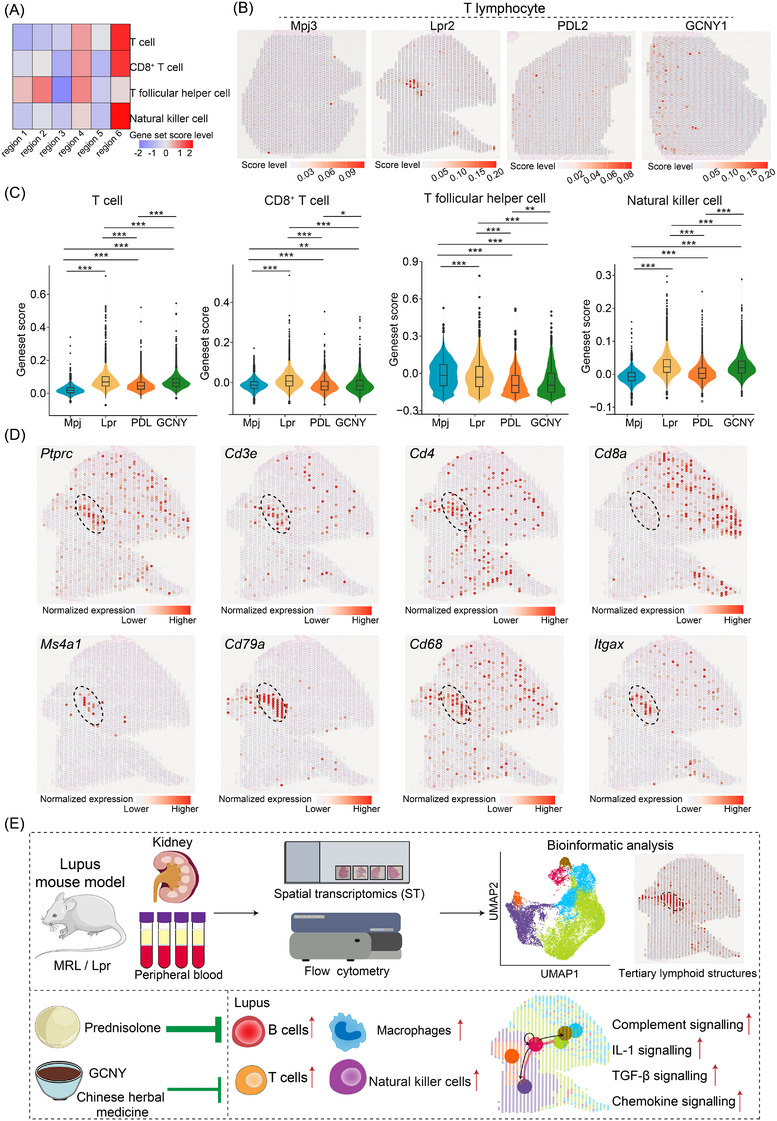
The characteristics of T lymphocyte and natural killer cell across groups. (A) Heatmap showing the signature score levels for T cells, CD4^+^ T cells, T follicular helper cells, and natural killer cells across regions. Red indicates higher score levels. (B) Spatial images depicting T lymphocyte enrichment scores in tissue slices, with red indicating higher levels. (C) Violin plots presenting signature score levels for T cells, CD4^+^ T cells, T follicular helper cells, and natural killer cells across groups, with percentiles indicated by the inside box plots (25%, 50% and 75%). (D) Spatial images displaying the expression levels of eight selected immune cell marker genes in the Lpr2 slice, with red indicating higher expression. (E) Schematic illustrating the highlights of this study.

Taking together, this study uncovers the regional characteristic in the LN kidney and captures a snapshot into TLS (Figure 4E). We found that GCNY treatment affected macrophage, B cell and T cell populations, though further research is needed to clarify the underlying mechanisms. These results offer insight to GCNY's therapeutic potential and provide a foundation for future clinical applications.

## AUTHOR CONTRIBUTIONS

**Yanjuan Chen**: Conceptualization; data curation; formal analysis; funding acquisition; investigation; methodology; project administration; resources; validation; visualization; funding acquisition; writing—original draft; writing—review and editing. **Yong Chen**: Conceptualization; investigation; methodology; resources; software; visualization; funding acquisition; writing—review and editing. **Xufa Yang**: Resources. **Xiaoping Hong, Dongzhou Liu and Zhenyou Jiang**: Supervision; funding acquisition; writing—review and editing.

## CONFLICT OF INTEREST STATEMENT

The authors declare no conflicts of interest.

## FUNDING INFORMATION

The study was supported by the National Natural Science Foundation of China (grant numbers: 82302030, 82460325), the Cultivation Project of Shenzhen People's Hospital (grant numbers: SYWGSLCYJ202203), the Sanming Project of Medicine in Shenzhen (grant numbers: SZSM202111006) and the Shenzhen Key Medical Discipline Construction Fund (grant numbers: SZXK011).

## ETHICS APPROVAL

The study design was approved by the Institutional Review Boards of the Shenzhen People's Hospital (approved no. LL‐KY‐2019504).

## Supporting information



Supporting Information

Supporting Information

## Data Availability

The data presented in the current study are available from the corresponding author upon reasonable request.
