# Supplementary material for: Spatial transcriptomics reveals regional characteristics of lupus nephritis in murine kidneys and immune response to prednisolone or Gancao Nourishing‐Yin decoction therapies
Source: Clin Transl Med. 2025 Feb 16;15(2):e70236. doi: 10.1002/ctm2.70236 (PMC11830562; doi:10.1002/ctm2.70236)
Supplement: Supplementary file 1 — Supporting Information [file CTM2-15-e70236-s001.docx]

**Spatial transcriptomics reveals regional characteristics of lupus nephritis in murine kidneys and immune response to prednisolone or Gancao Nourishing-Yin decoction therapies**

Yanjuan Chen ^1, †^, Yong Chen ^2, †^, Xufa Yang ^3^, Zhenyou Jiang ^4, #^, Dongzhou Liu ^1, #^, Xiaoping Hong ^3, #^

1. Department of Rheumatology and Immunology, The Second Clinical Medical College of Jinan University, Shenzhen People's Hospital, Shenzhen, Guangdong 518020, China.
2. Department of Rheumatology and Immunology, Affiliated Hospital of Zunyi Medical University, Zunyi, Guizhou 563000, China.
3. Department of Rheumatology and Immunology, The Second Clinical Medical College of Jinan University, The First Affiliated Hospital of Southern University of Science and Technology, Shenzhen People's Hospital, Shenzhen, Guangdong 518020, China.
4. Department of Microbiology and Immunology, School of Medicine, Jinan University, Guangzhou, Guangdong 510632, China.

^†^ Yanjuan Chen and Yong Chen contributed equally to this work.

^#^ Corresponding authors.

**Supplementary material**

**1. Supplementary** **methods and material**

**2. Supplementary results**

**3. Supplementary discussion**

**4. Supplementary references**

**5. Supplementary figures**

**1. Supplementary methods and material**

**1.1 Mice and treatments**

The MRL/Lpr mouse is well established for studying spontaneous LN.^1^ Six-week-old female Lpr mice and their control Mpj mice were procured from Changzhou Kavins Laboratory Animal Limited Liability Company (Production Permit No. SCXK(Su) 2021-0013) and housed at the SPF grape animal center. All experimental procedures were approved by the Ethics Committee of Shenzhen People's Hospital (No. LL-KY-2019504) and were performed in compliance with the Animal Welfare and Ethics guidelines of the Institutional Animal Care and Use Committee.

At 12 weeks of age, Lpr mice were randomly assigned to one of three treatment groups: distilled water (n = 3), PDL (n = 3), or prepared GCNY liquid (n = 3). Mice in the PDL group were treated with PDL at a dosage of 10 mg/kg per day for 4 weeks. Mice receiving GCNY were administered 12 g of crude medicine per kg body weight, equivalent to ten times the typical dosage for a 60 kg adult human. The GCNY formulation comprised seven herbs (Gancao, Ginseng, Yuzhu, Luohanguo, Pugongying, Ganjiang, and Dazao) in a ratio of 5:2:3:6:3:3:2. A total of 72 g of these raw herbs were boiled and simmered for 30 min. The herbs used in the GCNY preparation were provided by the Pharmacology Department of Shenzhen People's Hospital. After 4 weeks of treatment, all mice were anesthetized with an intramuscular injection of Zoletil (5 mg/100 g, Virbac, France).

**1.2 Preparation of renal single‐cell suspensions**

Fresh renal tissues were coarsely dissected using ophthalmic scissors and dissociated with the Kidney Tissue Dissociation Reagent (Miltenyi Biotec). The samples were transferred to a C tube (Miltenyi Biotec) and processed on a gentle MACS device (Miltenyi Biotec) following the kidney tissue program. The resulting suspension was filtered through a 70 μm cell strainer and washed twice with phosphate-buffered saline (PBS). Subsequently, cells were resuspended in RPMI 1640 culture medium supplemented with 5% fetal bovine serum (FBS).

**1.3 Flow cytometry analysis**

Peripheral blood samples of mice were collected from the abdominal aorta of mice and transferred to tubes containing ethylenediaminetetraacetic acid (EDTA). Blood samples and single-cell suspensions were stained for 15 minutes at 25°C with anti-B220 and anti-CD19 (BD Biosciences). After incubation, red blood cells were lysed with a lysing buffer (BD Biosciences) for 10 min. Cell fluorescence was acquired using a BD Arial II cell analyzer (BD Biosciences), and flow cytometry data were analyzed using FlowJo software (version 10.8.1).

**1.4 Bioinformatic analysis of spatial transcriptome data**

**1.4.1 ST Visium experiments**

Fresh kidney samples were sectioned to appropriate sizes, embedded in dry ice, and stored at -80℃ in sealed bags. Frozen tissues were sliced to 12 μm using a cryostat (Thermo Scientific), with the specimen head temperature maintained at -17°C and the blade temperature at -15°C. The sections were then placed in the capture area of the Visium spatial slice. Permeabilization of the slice was performed for 12 minutes according to the Visium Spatial Tissue Optimization protocol (10x Genomics). ST libraries were constructed following the Visium Spatial Gene Expression protocol.^2^

**1.4.2 Preprocessing of gene expression matrices**

The raw reads were initially preprocessed using the Space Ranger pipeline with GRCh38 reference genome to generate a spot-gene count matrix. Unique molecular identifier (UMI) counts were analyzed using the Seurat (version 4.1.1) package.^3^ Normalization was conducted with the SCTransform function. The Harmony algorithm was utilized to integrate all samples and eliminate batch effects^4^. Dimensionality reduction was conducted on the top 25 principal components, and unsupervised clustering analysis was visualized using a two-dimensional uniform manifold approximation and projection (UMAP).

**1.4.3 Multimodal intersection analysis (MIA)**

The MIA method^5,6^, utilizing the hypergeometric test, was applied to assess the cellular composition of spatial regions. The scRNA-seq dataset (accession No. GSE107585) from murine kidney tissues was retrieved from the Gene Expression Omnibus database.^7^ In the scRNA-seq data, renal cells were categorized into clusters such as proximal tubule cells, ascending loop of Henle cells, and endothelial cells.^7^ The enrichment score was determined by analyzing specific genes associated with each cell type, extracted from the scRNA-seq data, alongside corresponding genes from each cluster derived from the ST data.

**1.4.4 Deconvolution analysis**

Robust cell type decomposition (RCTD) is a deconvolution method that utilizes cell type profiles derived from scRNA-seq data to analyze mixtures of cell types, correcting for variations across sequencing technologies.^8^ Cell type decomposition on the ST slice was performed using the RCTD method with default parameters, referencing the scRNA-seq dataset (accession No. GSE107585). The primary subtype for each spot was identified as the one exhibiting maximum similarity.

**1.4.5 Identification of differentially expressed genes (DEGs)**

DEGs were identified using the FindMarkers and FindAllMarkers functions in Seurat.^3,9^ Genes with a fold change greater than 1.5 and an adjusted *P* value below 0.05 were classified as significant DEGs. The resulting DEGs are provided in the Supplementary table.

**1.4.6 Analysis of cell-cell communication**

The CellChat package^10^ was employed to identify related ligand-receptor pairs. Ligand–receptor pairs with a *P* value of less than 0.05 were considered to be significant interactions. The comparison mode in the Cellchat package was used to compare the cellular communication signals across the four groups. The signal enhancement in the Lpr group and attenuation in the PDL group were preferentially selected, followed by the signal attenuation in the GCNY. Finally, the signaling pathways related to lupus kidney were selected.

**1.4.7 Gene functional enrichment analysis**

Gene functional enrichment analyses of DEGs were performed using Metascape (http://metascape.org).^11^ Gene Set Enrichment Analysis (GSEA) was conducted using the ClusterProfiler package (version 4.14.3) ^12,13^ to compare Lpr versus Mpj, PDL versus Lpr, GCNY versus Lpr, GCNY versus PDL.

**1.4.8 Feature scoring of cell subtypes**

Feature gene sets for cell types in murine kidneys were sourced from the Cellmarker database.^14^ Signature scores of cell types were determined using the AddModuleScore function in the Seurat package with default parameters.

**1.5 Data statistics**

Statistical analysis was conducted using R (version 4.2.1). Graphical visualizations were created with the ggplot2 and pheatmap packages. The two-sided unpaired Wilcoxon test was employed for quantitative data that exhibited nonnormal distributions. A *P*-value of less than 0.05 was considered statistically significant.

**2. Supplementary results**

**2.1 Characterization of** **different regions in LN kidneys**

To investigate the transcriptional differences among regions, we analyzed DEGs, revealing distinct molecular signatures for each region. In region 1, upregulated DEGs such as *Napsa*, *Hao2*, and *Slc27a* are associated with metabolic, catabolic and biosynthetic processes (Figure S2, Table S1). Notably, increased HAO2 activity may enhance lipid catabolism and reduce lipid accumulation.^15^ In region 2, DEGs like *Calb1* and *S100g* are linked to calcium signaling, while region 3 displays elevated expression of *Mrps6*, *Wfdc15b*, and *Slc14a2*, which are involved in oxidative phosphorylation and renal functions (Figure S2A, B). Region 4 exhibits high expression of *Ren1*, *Cdkn1c*, *Plat*, *Podxl*, and *Nphs2* (Figure S2A, B). Here, *Plat* serves as an endothelial marker, whereas *Podxl* and *Nphs2* are specific to podocytes.^7,16^ In Region 5, *Aqp2* is prominently expressed, indicating the presence of collecting duct principal and transitional cells.^17^ Region 6 shows significant expression of immunoglobulin-related genes, such as *Ighg2c*, *Ighg2b*, and *Jchain*, suggesting an enrichment of plasma cells (Figure 2B). The upregulated DEGs in regions 4-6 are associated with hemostasis, regulation of locomotion, cell adhesion, and neutrophil degranulation, indicating overlapping molecular functions (Figure S2A, B). These shared markers imply that these regions may interact synergistically to mediate physiological processes (Figure S2C).

**3. Supplementary discussion**

ScRNA-seq technology has provided critical insights into the specific cell types and biomarkers involved in LN and has been valuable for exploring drug effects and therapeutic mechanisms.^7,18^ In 2019, Arazi et al. used scRNA-seq to map the immune cell landscape in LN-affected kidneys, revealing widespread expression of chemokine receptors CXCR4 and CX3CR1 in immune cells.^19^ However, the lack of spatial context makes it difficult to fully understand the interactions between cell populations in the kidney’s microenvironment.

Spatial transcriptomics (ST) addresses this limitation by providing spatial resolution and advances our understanding of tissue architecture and cell–cell interactions in sit.^20^ However, its application in LN kidneys has been limited. In this study, we employed ST to analyze renal tissues from lupus mice. Unsupervised clustering identified six distinct regions with heterogeneity and physiological roles. For instance, region 1 showed high expression of genes like *Napsa, Hao2,* and *Slc27a2*, which are involved in metabolic, catabolic, and biosynthetic processes. Spatial deconvolution, using the RCTD algorithm^8^, further detailed the cellular composition. Ten distinct cell populations were identified across ST spots, including proximal tubule, distal convoluted tubule, and ascending loop of Henle cells. These findings aligned with MIA results, showing region 1 as predominantly proximal tubule cells (95%), region 3 as mainly ascending loop of Henle cells (87%), and region 5 as largely endothelial cells (62%). Regions 4 and 6 exhibited the greatest variety of immune cell types, such as B lymphocytes and macrophages. These results offer insights into the distinct cellular landscapes of renal tissue regions.

Cell-cell interactions in LN kidneys involve diverse cell types and inflammatory pathways.^21^ Analysis of cell communications revealed activation of complement and TGF-β pathways between regions 4 and 6 in Lpr mice. Complement activation is associated with tissue damage in systemic lupus erythematosus, while TGF-β is linked to intrarenal nephritis activity in LN patients.^22,23^ Additionally, IL-1 signaling was found flowing from region 6 to region 3 and 4 in Lpr mice. Previous studies show elevated interleukin-1β (IL-1β) levels in Lpr mice compared to controls, and IL-1 inhibition has been shown to alleviate lupus-induced hyperalgesia.^24^ Chemokine signaling, including CCL19 and CXCL10, was also activated across regions 3, 4, and 6, both of which are correlated with disease severity.^25^ These results highlight key intercellular communication networks in the LN renal microenvironment.

Notably, we identified TLS enriched with immune cells in the Lpr2 slice. TLS is associated with severe tubulointerstitial inflammation, higher disease activity, and poor treatment response in LN patients.^26^ Similar structures have been observed in lupus-prone NZB/W mice, where TLS form organized networks with distinct T and B cell zones, adjacent dendritic cells, macrophages, and plasma cells.^27^ In the TLS of the Lpr2 slice, marker genes for T cells, B cells, plasma cells, macrophages, and dendritic cells were highly expressed. However, low *CD8a* expression suggests a limited presence of CD8^+^ T cells, warranting further investigation. Previous studies suggest that TLS formation in lupus is driven by elevated local chemokines like CXCL13 and CXCR5,^26,28^ which facilitate the recruitment of autoreactive B cells and promote lymphocyte aggregation in LN kidneys.^26^ Additionally, our analysis of cell-cell communication showed that TLS signals chemokines to other regions through ligand-receptor pairs such as Ccl8-Ccr5, Ccl3-Ccr5, Ccl5-Ccr5, Ccl27a-Ccr3, and Ccl8-Ccr2. These signaling molecules may serve as potential therapeutic targets for modulating TLS in LN treatment.

Glomerulonephritis is the key kidney lesion in systemic autoimmunity,^29^ but resolving the spatial distribution of immune cells in the kidney remains difficult due to their sparse presence.^30^ In this study, regions 4 and 6, enriched with immune cells and located in the renal cortex, were implicated in glomerular inflammation.

Traditional Chinese medicine (TCM) has shown promise in addressing LN-related kidney injury.^31,32^ With its broad pharmacological activities, TCM offers a potential alternative for managing chronic inflammatory diseases.^33^ Based on our previous research, GCNY demonstrated anti-inflammatory effects in a rheumatoid arthritis mouse model.^34^ GCNY-medicated serum also showed antioxidative effects in an H_2_O_2_-induced oxidative stress cell model.^35^ The percentage of regions 4 and 6 decreased in mice treated with PDL or GCNY, suggesting that GCNY may reduce kidney inflammation in LN.

Macrophages exhibit two main phenotypes: pro-inflammatory M1, which releases inflammatory cytokines, and anti-inflammatory M2, which facilitates tissue repair.^36^ Lupus flares are associated with an increased M1/M2 macrophage ratio, with M1 macrophages driving LN pathology.^37^ PDL treatment reduced the gene expression score for M1 macrophages, while GCNY slightly elevated the M2 macrophage score, indicating a potential shift towards an anti-inflammatory response. In GCNY-treated mice, lymphocyte activation was also downregulated, but innate immunity pathways remained active. These results suggest that both treatments reduce inflammation in LN macrophages, with PDL having a more pronounced immunosuppressive effect than GCNY.

Additionally, reductions in T cells, CD8^+^ T cells, T follicular helper cells, and natural killer cells were less pronounced in GCNY-treated mice compared to those treated with PDL. Flow cytometry also showed reduced peripheral CD19^+^ B cells and renal B220^+^ B cells in both treatment groups. PDL and GCNY also suppressed the complement, IL-1, and chemokine signaling pathways, supporting GCNY’s anti-inflammatory effects in LN. However, GCNY's immunosuppressive effect appears milder than PDL's. Traditional Chinese medicine (TCM), including our GCNY formula, tends to have a slower onset of action compared to the more rapid effects of chemical drugs like PDL.

Current technical limitations prevented single-cell resolution in our spatial sequencing. After this study, the 10x Genomics company officially released the Visium HD technology in early 2024. As a high-resolution spatial transcriptome, the upgraded Visium HD will provide more accurate spatial renal maps. In the current study, MIA and deconvolution analysis allowed us to capture preliminary features of kidney tissue in the LN mouse model. Despite the small sample size, the results of bioinformatics analysis and flow cytometry were consistent. Overall, this study offers valuable insights into LN pathogenesis and provides a theoretical basis for the therapeutic potential of GCNY in treating LN.

**4. Supplementary references**

1. Zhao XY, Li SS, He YX, et al. SGLT2 inhibitors alleviated podocyte damage in lupus nephritis by decreasing inflammation and enhancing autophagy. *Ann Rheum Dis.* 2023;82(10):1328-1340. <https://doi>: 10.1136/ard-2023-224242.

2. Wei R, He S, Bai S, et al. Spatial charting of single-cell transcriptomes in tissues. *Nat Biotechnol.* 2022;40(8):1190-1199. <https://doi>: 10.1038/s41587-022-01233-1.

3. Butler A, Hoffman P, Smibert P, Papalexi E, Satija R. Integrating single-cell transcriptomic data across different conditions, technologies, and species. *Nat Biotechnol.* 2018;36(5):411-420. <https://doi>: 10.1038/nbt.4096.

4. Korsunsky I, Millard N, Fan J, et al. Fast, sensitive and accurate integration of single-cell data with Harmony. *Nat Methods.* 2019;16(12):1289-1296. <https://doi>: 10.1038/s41592-019-0619-0.

5. Moncada R, Barkley D, Wagner F, et al. Integrating microarray-based spatial transcriptomics and single-cell RNA-seq reveals tissue architecture in pancreatic ductal adenocarcinomas. *Nat Biotechnol.* 2020;38(3):333-342. <https://doi>: 10.1038/s41587-019-0392-8.

6. Guo W, Zhou B, Yang Z, et al. Integrating microarray-based spatial transcriptomics and single-cell RNA-sequencing reveals tissue architecture in esophageal squamous cell carcinoma. *EBioMedicine.* 2022;84:104281. <https://doi>: 10.1016/j.ebiom.2022.104281.

7. Park J, Shrestha R, Qiu C, et al. Single-cell transcriptomics of the mouse kidney reveals potential cellular targets of kidney disease. *Science.* 2018;360(6390):758-763. <https://doi>: 10.1126/science.aar2131.

8. Cable DM, Murray E, Zou LS, et al. Robust decomposition of cell type mixtures in spatial transcriptomics. *Nat Biotechnol.* 2022;40(4):517-526. <https://doi>: 10.1038/s41587-021-00830-w.

9. Stuart T, Butler A, Hoffman P, et al. Comprehensive Integration of Single-Cell Data. *Cell.* 2019;177(7):1888-1902 e1821. <https://doi>: 10.1016/j.cell.2019.05.031.

10. Jin S, Guerrero-Juarez CF, Zhang L, et al. Inference and analysis of cell-cell communication using CellChat. *Nat Commun.* 2021;12(1):1088. <https://doi>: 10.1038/s41467-021-21246-9.

11. Zhou Y, Zhou B, Pache L, et al. Metascape provides a biologist-oriented resource for the analysis of systems-level datasets. *Nat Commun.* 2019;10(1):1523. <https://doi>: 10.1038/s41467-019-09234-6.

12. Subramanian A, Tamayo P, Mootha VK, et al. Gene set enrichment analysis: a knowledge-based approach for interpreting genome-wide expression profiles. *Proc Natl Acad Sci U S A.* 2005;102(43):15545-15550. <https://doi>: 10.1073/pnas.0506580102.

13. Wu T, Hu E, Xu S, et al. clusterProfiler 4.0: A universal enrichment tool for interpreting omics data. *Innovation (Camb).* 2021;2(3):100141. <https://doi>: 10.1016/j.xinn.2021.100141.

14. Hu C, Li T, Xu Y, et al. CellMarker 2.0: an updated database of manually curated cell markers in human/mouse and web tools based on scRNA-seq data. *Nucleic Acids Res.* 2023;51(D1):D870-D876. <https://doi>: 10.1093/nar/gkac947.

15. Zhang L, Yan Q, Lin M, et al. Investigation of ferroptosis-associated molecular subtypes and immunological characteristics in lupus nephritis based on artificial neural network learning. *Arthritis Res Ther.* 2024;26(1):126. <https://doi>: 10.1186/s13075-024-03356-z.

16. Yamaguchi J, Isnard P, Robil N, et al. PIK3CA inhibition in models of proliferative glomerulonephritis and lupus nephritis. *J Clin Invest.* 2024;134(15). <https://doi>: 10.1172/JCI176402.

17. Siegel CH, Sammaritano LR. Systemic Lupus Erythematosus: A Review. *JAMA.* 2024;331(17):1480-1491. <https://doi>: 10.1001/jama.2024.2315.

18. Van de Sande B, Lee JS, Mutasa-Gottgens E, et al. Applications of single-cell RNA sequencing in drug discovery and development. *Nat Rev Drug Discov.* 2023;22(6):496-520. <https://doi>: 10.1038/s41573-023-00688-4.

19. Arazi A, Rao DA, Berthier CC, et al. The immune cell landscape in kidneys of patients with lupus nephritis. *Nat Immunol.* 2019;20(7):902-914. <https://doi>: 10.1038/s41590-019-0398-x.

20. Rao A, Barkley D, Franca GS, Yanai I. Exploring tissue architecture using spatial transcriptomics. *Nature.* 2021;596(7871):211-220. <https://doi>: 10.1038/s41586-021-03634-9.

21. Roveta A, Parodi EL, Brezzi B, et al. Lupus Nephritis from Pathogenesis to New Therapies: An Update. *Int J Mol Sci.* 2024;25(16). <https://doi>: 10.3390/ijms25168981.

22. Fava A, Rao DA, Mohan C, et al. Urine Proteomics and Renal Single-Cell Transcriptomics Implicate Interleukin-16 in Lupus Nephritis. *Arthritis Rheumatol.* 2022;74(5):829-839. <https://doi>: 10.1002/art.42023.

23. Weinstein A, Alexander RV, Zack DJ. A Review of Complement Activation in SLE. *Curr Rheumatol Rep.* 2021;23(3):16. <https://doi>: 10.1007/s11926-021-00984-1.

24. Yan X, Maixner DW, Li F, Weng HR. Chronic pain and impaired glial glutamate transporter function in lupus-prone mice are ameliorated by blocking macrophage colony-stimulating factor-1 receptors. *J Neurochem.* 2017;140(6):963-976. <https://doi>: 10.1111/jnc.13952.

25. Dorner T, Tanaka Y, Dow ER, et al. Mechanism of action of baricitinib and identification of biomarkers and key immune pathways in patients with active systemic lupus erythematosus. *Ann Rheum Dis.* 2022;81(9):1267-1272. <https://doi>: 10.1136/annrheumdis-2022-222335.

26. Wang M, Rajkumar S, Lai Y, et al. Tertiary lymphoid structures as local perpetuators of organ-specific immune injury: implication for lupus nephritis. *Front Immunol.* 2023;14:1204777. <https://doi>: 10.3389/fimmu.2023.1204777.

27. Dorraji SE, Kanapathippillai P, Hovd AK, et al. Kidney Tertiary Lymphoid Structures in Lupus Nephritis Develop into Large Interconnected Networks and Resemble Lymph Nodes in Gene Signature. *Am J Pathol.* 2020;190(11):2203-2225. <https://doi>: 10.1016/j.ajpath.2020.07.015.

28. Jamaly S, Rakaee M, Abdi R, Tsokos GC, Fenton KA. Interplay of immune and kidney resident cells in the formation of tertiary lymphoid structures in lupus nephritis. *Autoimmun Rev.* 2021;20(12):102980. <https://doi>: 10.1016/j.autrev.2021.102980.

29. Kinloch AJ, Chang A, Ko K, et al. Vimentin is a dominant target of in situ humoral immunity in human lupus tubulointerstitial nephritis. *Arthritis Rheumatol.* 2014;66(12):3359-3370. <https://doi>: 10.1002/art.38888.

30. Benjamin K, Bhandari A, Kepple JD, et al. Multiscale topology classifies cells in subcellular spatial transcriptomics. *Nature.* 2024;630(8018):943-949. <https://doi>: 10.1038/s41586-024-07563-1.

31. Liu L, Zhang L, Li M. Application of herbal traditional Chinese medicine in the treatment of lupus nephritis. *Front Pharmacol.* 2022;13:981063. <https://doi>: 10.3389/fphar.2022.981063.

32. Ma Q, Xu M, Jing X, et al. Honokiol suppresses the aberrant interactions between renal resident macrophages and tubular epithelial cells in lupus nephritis through the NLRP3/IL-33/ST2 axis. *Cell Death Dis.* 2023;14(3):174. <https://doi>: 10.1038/s41419-023-05680-9.

33. Wang K, Yin J, Chen J, Ma J, Si H, Xia D. Inhibition of inflammation by berberine: Molecular mechanism and network pharmacology analysis. *Phytomedicine.* 2024;128:155258. <https://doi>: 10.1016/j.phymed.2023.155258.

34. Chen Y, Zhu XW, Lai WF, et al. Gancao Nourishing-Yin decoction combined with methotrexate in treatment of aging CIA mice: a study based on DIA proteomic analysis. *Chin Med.* 2023;18(1):9. <https://doi>: 10.1186/s13020-023-00709-9.

35. Chen Y, Wang B, Lai WF, et al. Chinese herbal formula (GCNY)-medicated serum alleviates peroxidation induced by H(2)O(2) in human microglial cells. *Front Neurosci.* 2022;16:990040. <https://doi>: 10.3389/fnins.2022.990040.

36. Zhao L, Tang S, Chen F, Ren X, Han X, Zhou X. Regulation of macrophage polarization by targeted metabolic reprogramming for the treatment of lupus nephritis. *Mol Med.* 2024;30(1):96. <https://doi>: 10.1186/s10020-024-00866-z.

37. Horuluoglu B, Bayik D, Kayraklioglu N, Goguet E, Kaplan MJ, Klinman DM. PAM3 supports the generation of M2-like macrophages from lupus patient monocytes and improves disease outcome in murine lupus. *J Autoimmun.* 2019;99:24-32. <https://doi>: 10.1016/j.jaut.2019.01.004.

**5. Supplementary figures**

**
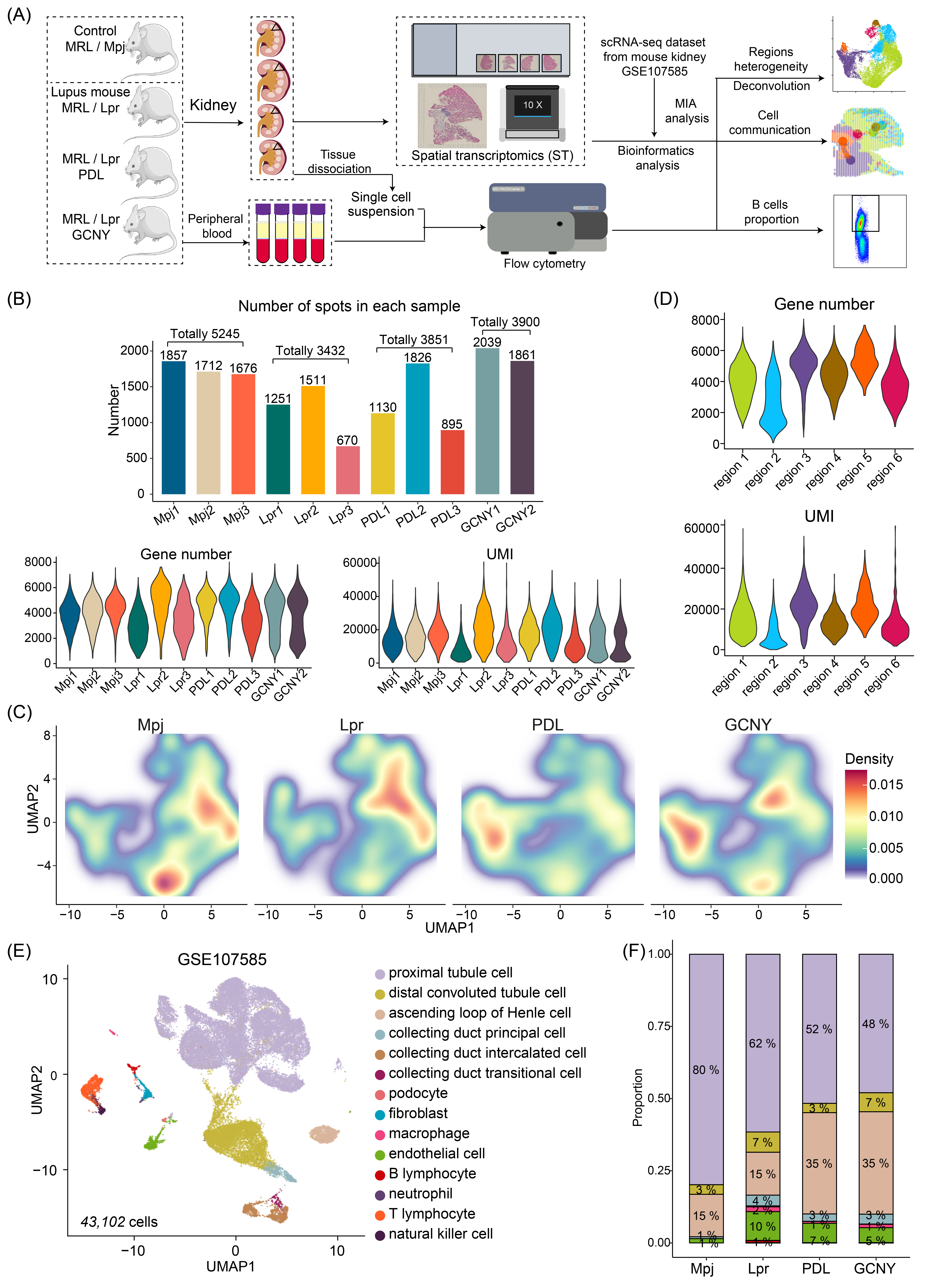
**

**Figure S1** Overview of the spatial transcriptomics in murine renal tissues. (A) Experimental design and analysis workflow. Renal tissues and peripheral blood were collected from MRL/Mpj (Mpj), MRL/Lpr (Lpr), MRL/Lpr with PDL treatment (PDL), and MRL/Lpr with GCNY treatment (GCNY) mice. Renal tissues were analyzed using ST via the 10x Genomics Visium platform. Flow cytometry analysis was conducted on a BD FACS Canto II system. Unsupervised clustering, MIA, and deconvolution analyses were performed in R studio software. (B) The bar plot above showing the number of spots across all samples. Violin plots below showing the number of detected genes and UMIs per spot across all samples. (C) UMAP density plots characterizing the distribution of spots across groups. (D)Violin plots presenting the number of genes and UMIs per spot for each region. (E) UMAP plot illustrating cell subtypes from scRNA-seq data of mouse kidneys, sourced from the GEO database (GSE107585). (F) Stacked bar plot showing the distribution of cell subtypes across groups.


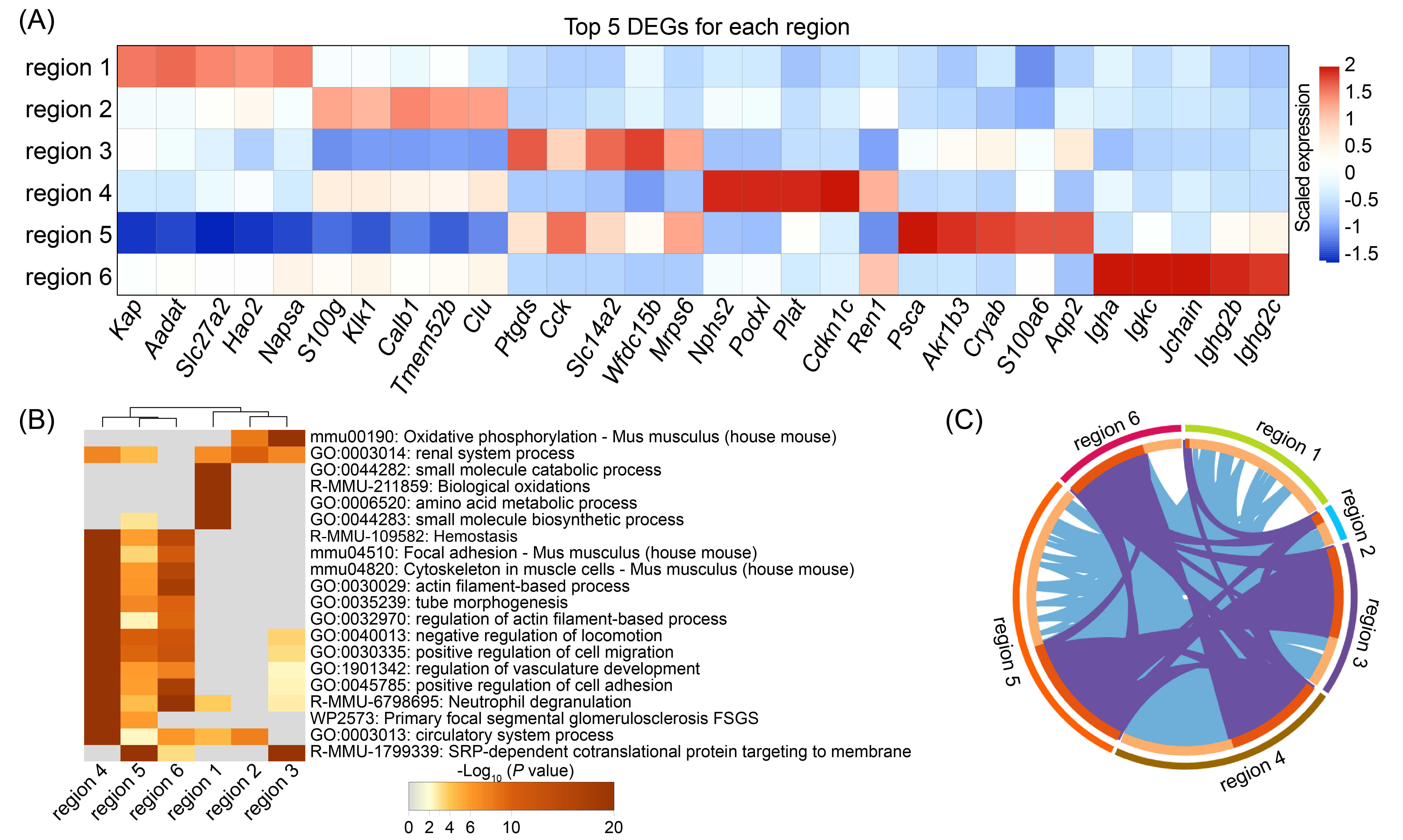


**Figure S2** Transcriptomic features of renal regions. (A) Heatmap of the average expression levels of the top 5 upregulated DEGs in each region, identified by comparing one region against others. Colors represent expression levels. (B) Heatmap of top enriched terms from Metascape analysis for upregulated DEGs in each region. Cells are colored by *P*-values, with grey indicating no significant enrichment (*P* < 0.05 was considered statistically significant). (C) Circle diagram illustrating the overlap of DEGs among regions. Purple lines connect genes shared between regions, with dark orange representing genes found in multiple lists and light orange indicating region-specific genes.


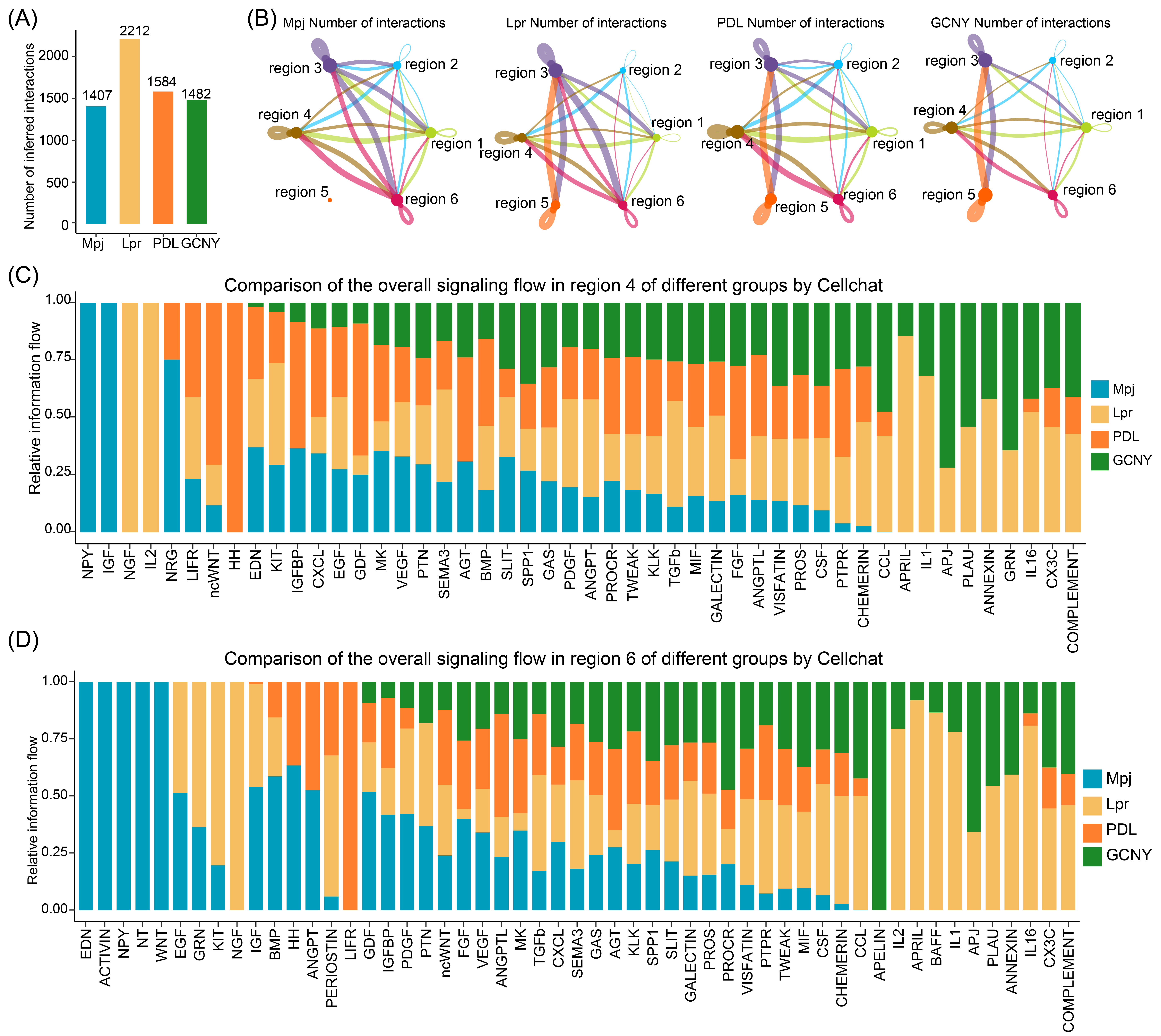


**Figure S3** Cellular communication analysis across groups. (A) Bar plot illustrating the number of interactions observed in the Mpj, Lpr, PDL, and GCNY groups. (B) Circle plots depicting the number of interactions between regions in each group, with edge width proportional to the number of interactions. (C) Stacked bar plot presenting the signaling pathways detected in region 4, ranked according to differences in overall information flow across the groups. Overall information flow was calculated by summing all communication probabilities within the inferred signaling networks. (D) Stacked bar plot showing the signaling pathways detected in region 6.

**
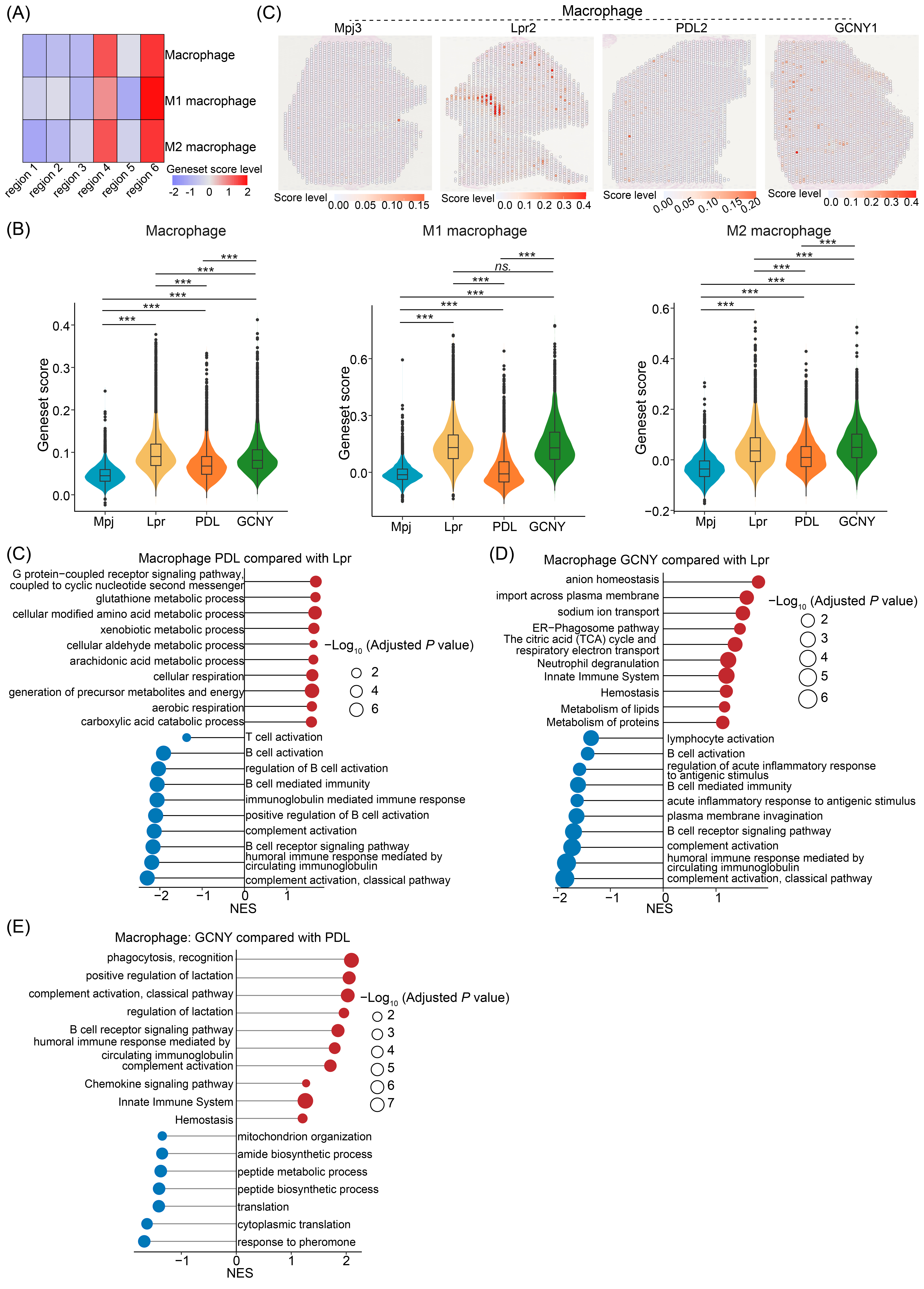
**

**Figure S4** Macrophage characteristics across groups. (A) Heatmap showing the signature score levels for macrophages, M1 macrophages, and M2 macrophages across regions. Red indicates higher score levels. (B) Violin plots displaying signature scores for macrophages, M1 macrophages, and M2 macrophages across groups, with percentiles represented by the inside box plots (25%, 50%, and 75%). (C) Spatial images showing macrophage enrichment scores across tissue slices, with H&E-stained sections overlaid. Red indicates higher enrichment levels. (C-E) Stick plots presenting the top activated (red) and suppressed (blue) signaling pathways in different comparisons, including PDL vs. Lpr, GCNY vs. Lpr, GCNY vs. PDL. Circle size represents the *P*-value from GSEA analysis. NES, normalized enrichment score.
